# No Evidence for an Association Between *DIP2B* Repeat Expansion and Neurological Disease

**DOI:** 10.1002/mds.70373

**Published:** 2026-05-28

**Authors:** Chia‐Ying Ko, Leon Schütz, Thomas Braun, Elena Buena‐Atienza, Nicolas Casadei, Danique Beijer, Ludger Schöls, Tobias B. Haack, Stephan Ossowski, Holger Hengel

**Affiliations:** ^1^ Institute of Medical Genetics and Applied Genomics University of Tübingen Tübingen Germany; ^2^ Department of Neurodegenerative Diseases, Center of Neurology and Hertie‐Institute for Clinical Brain Research University of Tübingen Tübingen Germany; ^3^ NGS Competence Center Tübingen University of Tübingen Tübingen Germany; ^4^ Center for Rare Diseases University Hospital Tübingen Tübingen Germany; ^5^ German Center for Neurodegenerative Diseases Tübingen Germany

**Keywords:** CGG, DIP2B, FRA12A, hypermethylation, long‐read sequencing, repeat expansion

Almost two decades ago, a CGG repeat expansion (RE) in the promoter region of *DIP2B* was described as the molecular basis of the folate‐sensitive fragile site FRA12A and associated with an autosomal dominant form of intellectual developmental disorder.^1^ This was based on two affected probands carrying ~270 to 300 CGG repeats and unaffected familial carriers with intermediate alleles of ~130 to 200 repeats. Analogous to fragile X syndrome, gene silencing induced by hypermethylation of REs exceeding a critical threshold was proposed as the pathogenic mechanism.

With the advent of long‐read sequencing, REs in *DIP2B* of variable size have been identified in several clinical contexts. To date, only one additional case of global developmental delay has been reported,^2^ while other cases include Lennox–Gastaut syndrome,^3^ Duchenne muscular dystrophy,^4^ cardiomyopathy,^5^ and two siblings with neurodevelopmental disability and a movement disorder (chorea, dystonia, and ataxia)^6^ (Table S1).

We used an in‐house database of 18,236 short‐read (srGS) and 950 long‐read (lrGS) genomes to screen for DIP2B CGG REs. Median repeat sizes [interquartile range] were similar between techniques (7 [7] and 8 [8.9]; Fig. 1A,B); however, numerous samples with >60 repeats (55 cases) were observed in srGS across probands with various disorders and healthy individuals. From these srGS cases, we selected the nine longest for lrGS validation, which confirmed our suspicion that CGG repeat lengths were frequently underestimated by srGS (Table S2).

**FIG. 1:**
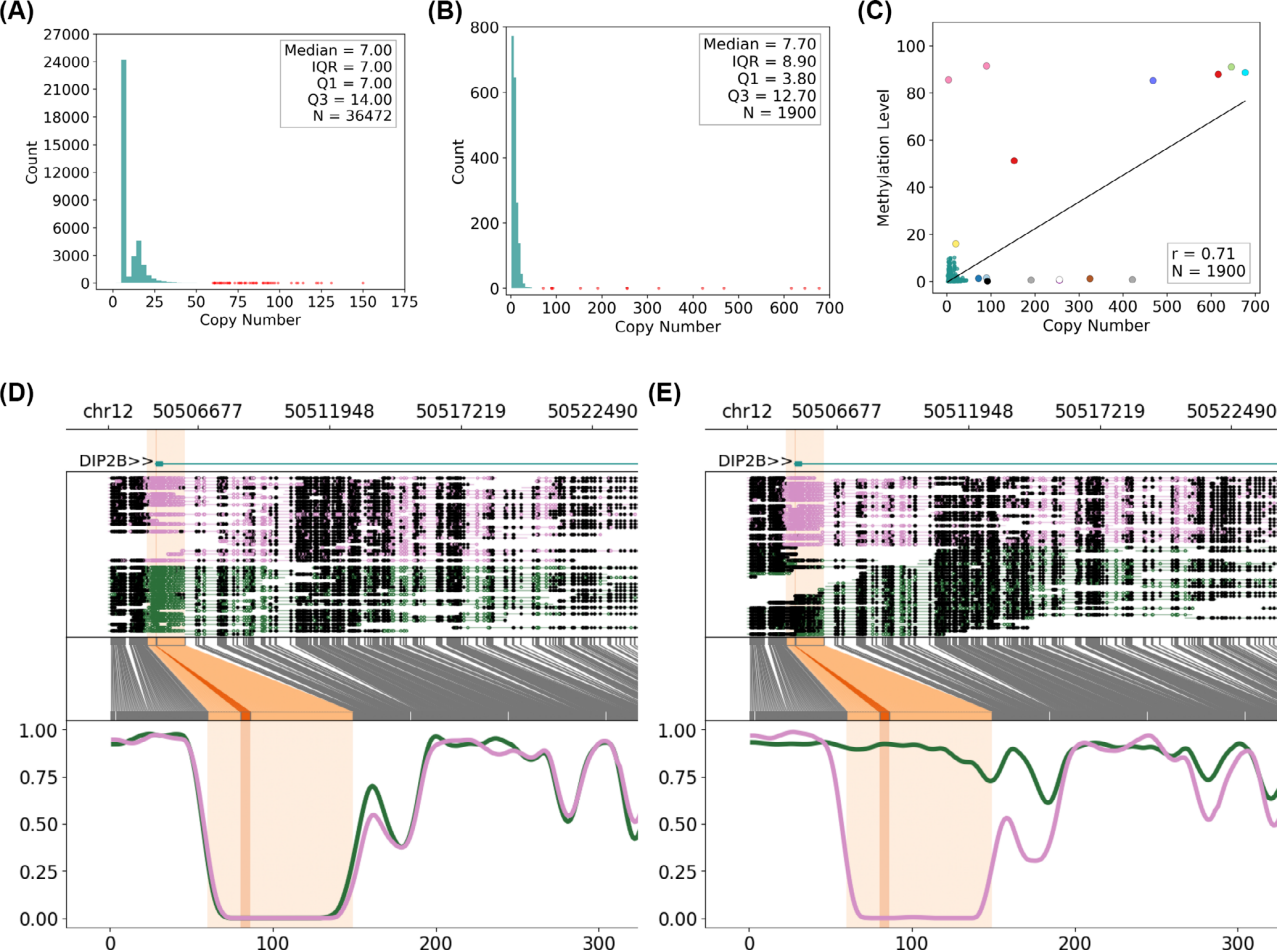
Analysis of *DIP2B* CGG repeat expansions in short‐read and long‐read genome sequencing cohorts. Histograms of predicted DIP2B CGG repeat (RE) lengths in short‐read (**A**) and long‐read (**B**) genomes. (**A**) Distribution of RE lengths across 18,236 short‐read genomes (36,472 alleles). (**B**) Distribution of RE lengths across 950 long‐read genomes (1900 alleles). The *x*‐axis indicates the predicted RE length per allele, and the *y*‐axis shows allele counts. Outliers were defined as values exceeding 6.5× the interquartile range (IQR) and are highlighted as red dots. In the short‐read dataset, 55 alleles (0.2%) exceeding 59.5 repeat copies were classified as outliers, whereas in the long‐read dataset, 15 alleles (0.8%) exceeding 70.55 repeat copies were identified as outliers. (**C**) Correlation analysis of long‐read genomes showing no strong association between DIP2B CGG repeat length and promoter hypermethylation (chr12:50504646–50506155). Methylation and repeat‐length outliers are color‐coded by phenotype: healthy (*red*), cerebellar ataxia (*gray*), amyotrophic lateral sclerosis (*white*), epileptic encephalopathy (*light green*), epilepsy (*black*), frontotemporal dementia (*blue‐purple*), nonneurological metabolic disease (*pink*), breast cancer (*cyan*), retinoschisis (*light blue*), rod‐cone dystrophy (*magenta*), optic atrophy (*brown*), Parkinson's disease (*dark blue*), and global developmental delay (*yellow*). (**D, E**) Exemplary methylation profiles of two samples with a DIP2B CGG repeat expansion. The repeat is located in the dark orange area (chr12:50505001–50505022). The promoter region is highlighted in light orange. Tracks show read‐level methylation for both alleles, CpG bin annotation, and smoothed modstat values, plotted across CpG bins and genomic coordinates (*x*‐axis), with modstat values representing the log ratio of methylated to unmethylated states on the *y*‐axis. One sample shows no evidence of promoter hypermethylation (**D**), whereas the other displays increased methylation at the expanded allele (**E**). N, total number of alleles analyzed; r, Pearson's correlation coefficient. [Color figure can be viewed at wileyonlinelibrary.com]

From these validated lrGS cases and the lrGS database, we established a cohort of 14 cases with lrGS data harboring long to very long *DIP2B* REs (90–677 repeats) to assess their potential clinical impact. Our cohort spanned a broad spectrum of disease groups and healthy individuals (Table S3). No common phenotypic pattern could be identified. Among individuals with the longest repeats, there were healthy probands (152 and 616 repeats) and a patient with breast carcinoma (677 repeats). Other individuals had rod‐cone dystrophy or heterogeneous neurological disorders. Detailed analysis of the repeat sequences further showed cases with pure and impure motifs, motif changes, and broad repeat‐length distributions, indicative of somatic mosaicism (Table S3, Supporting Information Figs. S1–S14).

We next investigated the correlation between promoter (hyper)methylation and repeat length and found that, although some probands with longer repeats showed hypermethylation of the promoter region, several others did not (Fig. 1C,D). Promoter hypermethylation was also observed in several probands without RE. Hypermethylated samples came from individuals with various neurological and nonneurological disorders, as well as from healthy individuals. Because constraint metrics in gnomad v4.1.1 indicated moderate to high intolerance against loss‐of‐function variants, we also screened our in‐house database (42,356 srGS or exomes) for loss‐of‐function variants in *DIP2B* and identified 40 heterozygous carriers, again without evidence of a shared phenotypic pattern, arguing against haploinsufficiency of *DIP2B* as a disease‐causing mechanism. In summary, based on the largest cohort of individuals with expanded *DIP2B* alleles reported to date, we found no evidence to support an association between *DIP2B* CGG RE, *DIP2B* promoter hypermethylation, or loss‐of‐function variants and neurological disease.

## Author Roles

1. Research Project: A. Conception, B. Organization, C. Execution; 2. Statistical Analysis: A. Design, B. Execution, C. Review and Critique; 3. Manuscript: A. Writing of First Draft, B. Review and Critique.

C.‐Y.K.: 1C, 2B, 3A, 3C.

L.S.: 1C, 2C, 3C.

T.B.: 1B, 2C, 3C.

E.B.‐A.: 1B, 1C, 3C.

N.C.: 1B, 1C, 3C.

D.B.: 1B, 2C, 3C.

L.S.: 1C, 2C, 3C.

T.B.H.: 1C, 2C, 3C.

S.O.: 1A, 1B, 2A, 2C, 3C.

H.H.: 1A, 1B, 1C, 2A, 2C, 3A, 3B.

## Financial Disclosures and Conflicts of Interest

C.‐Y.K. was supported by Hertie‐Network of Excellence in Clinical Neuroscience. E.B.A. performed next‐generation sequencing (NGS) sequencing with the support of the Deutsche Forschungsgemeinschaft (DFG)‐funded NGS Competence Center Tübingen (INST 37/1049‐1). N.C. performed NGS sequencing with the support of the DFG‐funded NGS Competence Center Tübingen (INST 37/1049‐1). D.B. was supported by a Humboldt Research Fellowship for Postdocs and the Hertie‐Network of Excellence in Clinical Neuroscience. L.S. received funding by the German Ministry of Education and Research (BMBF grants 01GM2209F, 01GM2210A, and 01ED2503B) and the DFG (grant SCHO754/8‐1). T.B.H. received funding from the European Commission (Recon4IMD – GAP‐101080997) and the DFG (EJP‐RD Artemis: 542553983). S.O. was supported by the DFG (OS 647/1‐1). H.H. was supported by the DFG (HE 8803/1‐1). Author disclosures are available in the Supporting Information.

## Supporting information


**Figure S1.** Motif waterfall plot for proband 1. Long‐read sequences (n = 28) are ordered by total repeat expansion length. Flanking anchor sequences (black) are aligned to the insertion boundaries. The *x*‐axis represents the relative position (bp) within the expansion. Different repeat motifs are color coded within the repeat. Motif waterfall plots were generated using a custom Python‐based visualization pipeline. The tool utilized the Pysam library to parse BAM alignment files, extracting raw sequence data and CIGAR‐string information for individual reads at the target locus. Reads were anchored using flanking reference sequences as fixed points to ensure structural comparability. This established the insertion site as the zero point (0 bp), from which the internal expansion and motif segments were rendered using the Matplotlib library. Motifs were collapsed into their canonical cyclic forms before visualization to maintain color consistency across reads. The motif changes and somatic mosaicism shown in Figures S2–S14 should be validated in future studies using an orthogonal method.
**Figure S2.** Motif waterfall plot for proband 2. Long‐read visualization (n = 32) sorted by total expansion length. Flanking anchors (black) are aligned to the insertion boundaries. The *x*‐axis indicates the relative position (bp) within the expansion.
**Figure S3.** Motif waterfall plot for proband 3. Long‐read visualization (n = 16) sorted by total expansion length. Flanking anchors (black) are aligned to the insertion boundaries. The *x*‐axis indicates the relative position (bp) within the expansion.
**Figure S4.** Motif waterfall plot for proband 4. Long‐read visualization (n = 30) sorted by total expansion length. Flanking anchors (black) are aligned to the insertion boundaries. The x‐axis indicates the relative position (bp) within the expansion.
**Figure S5.** Motif waterfall plot for proband 5. Long‐read visualization (n = 16) sorted by total expansion length. Flanking anchors (black) are aligned to the insertion boundaries. The *x*‐axis indicates the relative position (bp) within the expansion.
**Figure S6.** Motif waterfall plot for proband 6. Long‐read visualization (n = 35) sorted by total expansion length. Flanking anchors (black) are aligned to the insertion boundaries. The *x*‐axis indicates the relative position (bp) within the expansion.
**Figure S7.** Motif waterfall plot for proband 7. Long‐read visualization (n = 13) sorted by total expansion length. Flanking anchors (black) are aligned to the insertion boundaries. The *x*‐axis indicates the relative position (bp) within the expansion.
**Figure S8.** Motif waterfall plot for proband 8. Long‐read visualization (n = 27) sorted by total expansion length. Flanking anchors (black) are aligned to the insertion boundaries. The *x*‐axis indicates the relative position (bp) within the expansion.
**Figure S9.** Motif waterfall plot for proband 10. Long‐read visualization (n = 16) sorted by total expansion length. Flanking anchors (black) are aligned to the insertion boundaries. The *x*‐axis indicates the relative position (bp) within the expansion.
**Figure S10.** Motif waterfall plot for proband 11. Long‐read visualization (n = 10) sorted by total expansion length. Flanking anchors (black) are aligned to the insertion boundaries. The *x*‐axis indicates the relative position (bp) within the expansion.
**Figure S11.** Motif waterfall plot for proband 12. Long‐read visualization (n = 18) sorted by total expansion length. Flanking anchors (black) are aligned to the insertion boundaries. The *x*‐axis indicates the relative position (bp) within the expansion.
**Figure S12.** Motif waterfall plot for proband 13. Long‐read visualization (n = 23) sorted by total expansion length. Flanking anchors (black) are aligned to the insertion boundaries. The *x*‐axis indicates the relative position (bp) within the expansion.
**Figure S13.** Motif waterfall plot for proband 14. Long‐read visualization (n = 46) sorted by total expansion length. Flanking anchors (black) are aligned to the insertion boundaries. The x‐axis indicates the relative position (bp) within the expansion.
**Figure S14.** Motif waterfall plot for proband 15. Long‐read visualization (n = 32) sorted by total expansion length. Flanking anchors (black) are aligned to the insertion boundaries. The *x*‐axis indicates the relative position (bp) within the expansion.

**Data S1** Supporting Information Methods.

**Table S1.** Previously reported cases with *DIP2B* repeat expansions.
**Table S2.** Estimated lengths of elongated repeat expansions in short‐read and long‐read genomes.
**Table S3.** Repeat length, methylation, and phenotype of probands with expanded CGG repeats.

## Data Availability

The data that support the findings of this study are available from the corresponding author upon reasonable request.

## References

[1] Winnepenninckx B , Debacker K , Ramsay J , Smeets D , Smits A , FitzPatrick DR , Kooy RF . CGG‐repeat expansion in the DIP2B gene is associated with the fragile site FRA12A on chromosome 12q13.1. Am J Hum Genet 2007;80(2):221–231. 10.1086/510800 PMC1785358 17236128

[2] Cheung WA , Johnson AF , Rowell WJ , et al. Direct haplotype‐resolved 5‐base HiFi sequencing for genome‐wide profiling of hypermethylation outliers in a rare disease cohort. Nat Commun 2023;14(1):3090. 10.1038/s41467-023-38782-1 PMC10226990 37248219

[3] Qaiser F , Sadoway T , Yin Y , et al. Genome sequencing identifies rare tandem repeat expansions and copy number variants in Lennox‐Gastaut syndrome. Brain Commun 2021;3(3):fcab207. 10.1093/braincomms/fcab207 PMC8491034 34622207

[4] Folland C , Ganesh V , Weisburd B , et al. Transcriptome and genome analysis uncovers a DMD structural variant: a case report. Neurol Genet 2023;9(2):e200064. 10.1212/NXG.0000000000200064 PMC10117699 37090938

[5] Mitina A , Khan M , Lesurf R , et al. Genome‐wide enhancer‐associated tandem repeats are expanded in cardiomyopathy. EBioMedicine 2024;101:105027. 10.1016/j.ebiom.2024.105027 PMC10944212 38418263

[6] Théberge ET , Durbano K , Demailly D , et al. Disco‐interacting protein 2 homolog B CGG repeat expansion in siblings with neurodevelopmental disability and progressive movement disorder. Move Disord 2025;40(3):567–578. 10.1002/mds.30101 39854091

