# Supplementary material for: No Evidence for an Association Between DIP2B Repeat Expansion and Neurological Disease
Source: Mov Disord. 2026 May 28;41(9):2529–31. doi: 10.1002/mds.70373 (PMC13602277; doi:10.1002/mds.70373)
Supplement: Supplementary file 1 [file MDS-41-2529-s002.docx]

**Supplementary Methods**

**1. *DIP2B* repeat expansion (RE) screening of short-read (sr) cohort**

**1.1 Study oversight, cohort composition, and data generation**

Retrospect local data re-analysis of the investigated cohort was approved by the Ethics Committee of the University of Tübingen (#066/2021BO2). A total of 18,236 sr genome sequencing (GS) datasets from an in-house database were analyzed. This in-house srGS cohort is derived from our diagnostic pipeline and includes individuals with a broad range of clinical indications. The most frequent indications are neurological and neurodevelopmental disorders (36%), followed by neoplasms (19.5%) and diseases of the visual system (19.5%). A total of 9.7% of individuals are classified as healthy, the majority of whom are unaffected parents or relatives included in a diagnostic context.
Genomic DNA was extracted from blood leukocytes. Libraries were prepared using the TruSeq DNA PCR-Free Library Prep Kit (Illumina) and sequenced on a NovaSeq 6000 system as 150-bp paired-end reads, with an average 45× coverage.

**1.2 Basic analysis pipeline**

srGS data were processed using the megSAP pipeline. Read alignment to GRCh38 reference genome was performed using bwa-mem2.

**1.3 Repeat calling**

Repeat expansions in srGS were identified using ExpansionHunter (v5.0.0).

**2. DIP2B repeat expansion (RE) screening of long-read (lr) cohort**

**2.1 Cohort composition and library preparation**

A total of 950 lrGS datasets from an in-house database were used to establish the background distribution and identify DIP2B RE outliers. This long-read cohort is derived from multiple research projects and includes the following distribution of phenotype groups and healthy individuals: neurological and neurodevelopmental disorders (68%), healthy individuals (16.9%), diseases of the visual system (6.3%), and a small proportion of other heterogeneous conditions.
Genomic DNA was mainly extracted from blood leukocytes. Genomic DNA was sheared with Megaruptor 3 (Diagenode) and libraries were prepared using the SQK-LSK114 Sequencing Kit V14 (Oxford Nanopore Technologies). Genomic DNA and libraries were quantified using the dsDNA High Sensitivity assay on a Qubit 3 fluorometer (Thermo Fisher) and the integrity was assessed with Genomic DNA 165 kb Analysis Kit on a FemtoPulse (Agilent). Sequencing was performed with PromethION R10.4.1 flow cells.

**Suppelmentary Methods Figure 1: Composition of the lrGS cohort.**
Pie chart of the distribution of disease groups and healthy controls in 950 sequenced long-read genomes.

**2.2 Basic analysis pipeline**

Basic processing of lr GS data was performed using the megSAP pipeline. megSAP is publicly available on GitHub (https://github.com/imgag/megSAP; accessed January 22, 2026). Reads alignment against to GRCh38 reference genome was performed using minimap2. Methylation information was subsequently extracted using modkit.

**2.3 Repeat calling**

Screening of the *DIP2B* CGG repeat locus (chr12:50,505,001-50,505,022) across the long-read cohort was carried out using Straglr (v1.5.3), followed by read reclustering with the add-on tool StraglrOn (v0.2.2) using the '--altclust' parameter.

**2.4 Methylation profiling**

Methylation profiles were visualized using MethylArtist (https://github.com/adamewing/methylartist), with calculations based on modkit outputs. Read-level methylation for individual cytosines was aggregated across a 1.5 kb genomic region flanking the *DIP2B* CGG repeat locus (chr12:50,504,646-50,506,155) for all lrGS. For correlation analyses, longer alleles were assigned higher methylation levels. An exception was made for the 15 RE outliers, which were manually inspected for 5mC base modifications in BAM files using Integrative Genomics Viewer (IGV) to ensure accurate assignment.

**2.5 Manual curation of repeat length**

Misreporting of expansion copy numbers, including both over- and underestimation by repeat calling tools was observed. This variability is attributable to the presence of reads spanning a wide range of repeat lengths within individual alleles, as well as to the occurrence of impure repeat motifs. Consequently, manual curation was undertaken for the 15 identified repeat expansion outliers. Repeat copy numbers were redefined as the median insertion size derived from reads showing an insertion in IGV, as summarized in Table S3. Pure CGG repeats and full-length impure motifs were quantified separately and are reported in Table S3.

**3. Resequencing of selected SR GS**

Nine cases with REs from the srGS cohort with the longest estimated *DIP2B* CGG were selected and re-sequenced using lrGS for validation of the predicted repeat lengths.

**4. Loss-of-function (LoF) variant screening**

Predicted LoF variants in *DIP2B*, including stop-gain, stop-loss, frameshift, splice-acceptor and splice-donor variants, were screened using GSvar (https://github.com/imgag/ngs-bits/blob/master/doc/GSvar/index.md) The analysis was carried out across 42,356 in-house SR GS and exome datasets, with a variant quality score cutoff of 20.
