# Supplementary material for: No Evidence for an Association Between DIP2B Repeat Expansion and Neurological Disease
Source: Mov Disord. 2026 May 28;41(9):2529–31. doi: 10.1002/mds.70373 (PMC13602277; doi:10.1002/mds.70373)
Supplement: Supplementary file 2 [file MDS-41-2529-s003.docx]

**Supplementary Figures**

**Supplementary Figure S1:** Motif Waterfall plot for Proband 1. Long-read sequences (n = 28) are ordered by total repeat expansion length. Flanking anchor sequences (black) are aligned to the insertion boundaries. The x-axis represents the relative position (bp) within the expansion. Different repeat motifs are color coded within the repeat.

Motif waterfall plots were generated using a custom Python-based visualization pipeline. The tool utilized the Pysam library to parse BAM alignment files, extracting raw sequence data and CIGAR-string information for individual reads at the target locus. To ensure structural comparability, reads were anchored using flanking reference sequences as fixed points. This established the insertion site as the zero point (0 bp), from which the internal expansion and motif segments were rendered using the Matplotlib library. To maintain color consistency across reads, motifs were collapsed into their canonical cyclic forms prior to visualization.

The motif changes and somatic mosaicism shown in the following supplementary figures should be validated in future studies using an orthogonal method.

**Supplementary Figure S2:** Motif waterfall plot for Proband 2. Long-read visualization (n=32) sorted by total expansion length. Flanking anchors (black) are aligned to the insertion boundaries. The x-axis indicates the relative position (bp) within the expansion.

Expansion size in bp

 **Supplementary Figure S3**: Motif waterfall plot for Proband 3. Long-read visualization (n=16) sorted by total expansion length. Flanking anchors (black) are aligned to the insertion boundaries. The x-axis indicates the relative position (bp) within the expansion.

 **Supplementary Figure S4**: Motif waterfall plot for Proband 4. Long-read visualization (n=30) sorted by total expansion length. Flanking anchors (black) are aligned to the insertion boundaries. The x-axis indicates the relative position (bp) within the expansion.

 **Supplementary Figure S5**: Motif waterfall plot for Proband 5. Long-read visualization (n=16) sorted by total expansion length. Flanking anchors (black) are aligned to the insertion boundaries. The x-axis indicates the relative position (bp) within the expansion.

 **Supplementary Figure S6**: Motif waterfall plot for Proband 6. Long-read visualization (n=35) sorted by total expansion length. Flanking anchors (black) are aligned to the insertion boundaries. The x-axis indicates the relative position (bp) within the expansion.

 **Supplementary Figure S7**: Motif waterfall plot for Proband 7. Long-read visualization (n=13) sorted by total expansion length. Flanking anchors (black) are aligned to the insertion boundaries. The x-axis indicates the relative position (bp) within the expansion.

 **Supplementary Figure S8**: Motif waterfall plot for Proband 8. Long-read visualization (n=27) sorted by total expansion length. Flanking anchors (black) are aligned to the insertion boundaries. The x-axis indicates the relative position (bp) within the expansion.

 **Supplementary Figure S9**: Motif waterfall plot for Proband 10. Long-read visualization (n=16) sorted by total expansion length. Flanking anchors (black) are aligned to the insertion boundaries. The x-axis indicates the relative position (bp) within the expansion.

 **Supplementary Figure S10**: Motif waterfall plot for Proband 11. Long-read visualization (n=10) sorted by total expansion length. Flanking anchors (black) are aligned to the insertion boundaries. The x-axis indicates the relative position (bp) within the expansion.

 **Supplementary Figure S11**: Motif waterfall plot for Proband 12. Long-read visualization (n=18) sorted by total expansion length. Flanking anchors (black) are aligned to the insertion boundaries. The x-axis indicates the relative position (bp) within the expansion.

 **Supplementary Figure S12**: Motif waterfall plot for Proband 13. Long-read visualization (n=23) sorted by total expansion length. Flanking anchors (black) are aligned to the insertion boundaries. The x-axis indicates the relative position (bp) within the expansion.

 **Supplementary Figure S13**: Motif waterfall plot for Proband 14. Long-read visualization (n=46) sorted by total expansion length. Flanking anchors (black) are aligned to the insertion boundaries. The x-axis indicates the relative position (bp) within the expansion.

 **Supplementary Figure S14**: Motif waterfall plot for Proband 15. Long-read visualization (n=32) sorted by total expansion length. Flanking anchors (black) are aligned to the insertion boundaries. The x-axis indicates the relative position (bp) within the expansion.
